# Biomarkers of biological age as predictors of COVID-19 disease severity

**DOI:** 10.18632/aging.103052

**Published:** 2020-04-08

**Authors:** Gordan Lauc, David Sinclair

**Affiliations:** 1 University of Zagreb Faculty of Pharmacy and Biochemistry and Genos Glycoscience Research Laboratory, Zagreb, Croatia; 2 Paul F. Glenn Center for Biology of Aging Research, Department of Genetics, Blavatnik Institute, Harvard Medical School, Boston, MA 02115, USA; 3 Laboratory for Ageing Research, Department of Pharmacology, School of Medical Sciences, The University of New South Wales, Sydney, NSW 2052, Australia

**Keywords:** COVID-19, biomarkers of biological age, biological age

Early epidemiological studies suggest the most important predictor of severity of COVID-19 disease course is age. Pre-existing conditions, including diabetes, CVD, hypertension, obesity and other consequences of an unhealthy lifestyle are also associated with increased mortality, indicating that the biological age is more relevant than the chronological age. Because a reliable COVID-19 vaccine is unlikely to available before the maximal infection of COVID-19 has occurred, it is essential to establish reliable tools for patient stratification and identification of individuals at high risk of severe disease.

A number of biomarkers aimed at objective estimation of biological age have been developed in the past several years, the most prominent ones being the epigenetic clock and the glycan clock. A key feature of a good biomarker of biological age is that the difference between chronological and biological age should correlate with known biomarkers of unhealthy lifestyle and that increased biological age should predict future disease development. The original epigenetic clock relied, in part, on chronological age, so several alternative epigenetic clocks, such as the GrimAge methylation clock, were developed. This has been demonstrated for both methylation and glycans. The difference between glycan age and chronological age associates with biomarkers of unhealthy lifestyle [1], while changes in glycans predict future diabetes and cardiovascular events [2]. Several different epigenetic clocks were recently also ﻿shown to predict prevalence and incidence of leading causes of death and disease [3].

Glycans, or polysaccharides, are carbohydrate-based polymers that regulate a variety of processes, including immunity [4]. In fact, glycan diversity represents one of the main defenses of all higher organisms against pathogens, and the repertoire of glycans changes with age, especially in the age ranges that are most susceptible to SARS-CoV2. Furthermore, both the SARS-Cov-2 virus and its principal cellular target ACE2 are known to be highly glycosylated [5], a pattern that likely changes with age. Recent study analysed site-specific N-linked glycosylation of MERS and SARS S glycoproteins, indicating that each of these glycosylation sites can be occupied by up to ten different glycans (called glycoforms), which greatly extends epitope diversity [6].

Glycans are the primary molecular basis inter-individual differences within the human population, including the ABO blood groups. Furthermore, glycans are one of the principal regulators of antibody effector functions and many other aspects of the immune system. Based on these and other findings, we believe that glycans should be in the focus of biomarker discovery in COVID-19 cases. Since glycans are structurally complex and their analysis is technically challenging, until recently they were largely ignored by clinical researchers. However, the situation changed dramatically in the last few years and through the Human Glycome Project over 100,000 glycome profiling has been performed, resulting in many prominent discoveries of promising glycan biomarkers.

**Figure 1:**
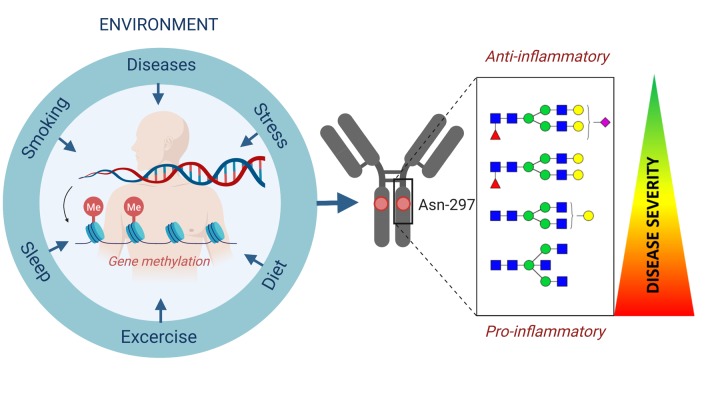
Information from genetic, epigenetic and direct environmental factors integrate at the level of protein glycosylation and result in inter-individual differences in both expression of surface antigens and regulation of the immune system.

Glycans are inherited as complex traits and also affected by epigenetic memory of environmental factors [7]. Environmental factors such as smoking and diabetes could alter the glycan repertoire directly or by increasing biological (Figure 1) [2,8].

Reports from Italy and US indicate that in case of insufficient ICU capacity triage of COVID-19 patients is based on subjectively defined criteria that are not based on strong data. At present, we still do not understand the molecular basis of severe COVID-19 symptoms, so research is urgently needed to identify biomarkers that could enable early identification of high-risk individuals. Therefore, it is of utmost importance to biobank large number of plasma samples of both severe and mild cases, so that modern profiling technologies can be used to identify molecular risk factors during this and for future outbreaks. We understand that our colleagues at the frontlines of this pandemics are overwhelmed with saving lives, but biobanking samples has a potential to save many more lives in the future.
